# Lessons learned for surveillance system strengthening through capacity building and partnership engagement in post-Ebola Guinea, 2015–2019

**DOI:** 10.3389/fpubh.2022.715356

**Published:** 2022-08-11

**Authors:** Jennifer J. Hemingway-Foday, Boubacar Ibrahima Diallo, Salomon Compaore, Souleymane Bah, Sakoba Keita, Ibrahima Telly Diallo, Lise D. Martel, Claire J. Standley, Mariama B. Bah, Marlyatou Bah, Djiguiba Camara, Almamy K. Kaba, Lamine Keita, Moussa Kone, Eileen Reynolds, Ousmane Souare, Kristen B. Stolka, Samuel Tchwenko, Abdoulaye Wone, Mary Claire Worrell, Pia D. M. MacDonald

**Affiliations:** ^1^RTI International, Durham, NC, United States; ^2^RTI International, Conakry, Guinea; ^3^International Organization for Migration, Conakry, Guinea; ^4^International Medical Corps, Conakry, Guinea; ^5^Ministry of Health, Conakry, Guinea; ^6^Division of Global Health Protection, Centers for Disease Control and Prevention, Atlanta, GA, United States; ^7^Center for Global Health Science and Security, Georgetown University, Washington, DC, United States; ^8^Department of Epidemiology, Gillings School of Public Health, University of North Carolina, Chapel Hill, NC, United States

**Keywords:** surveillance, Global Health Security Agenda, partnership, collaboration, capacity building

## Abstract

The 2014–2016 Ebola outbreak in Guinea revealed systematic weaknesses in the existing disease surveillance system, which contributed to delayed detection, underreporting of cases, widespread transmission in Guinea and cross-border transmission to neighboring Sierra Leone and Liberia, leading to the largest Ebola epidemic ever recorded. Efforts to understand the epidemic's scale and distribution were hindered by problems with data completeness, accuracy, and reliability. In 2017, recognizing the importance and usefulness of surveillance data in making evidence-based decisions for the control of epidemic-prone diseases, the Guinean Ministry of Health (MoH) included surveillance strengthening as a priority activity in their post-Ebola transition plan and requested the support of partners to attain its objectives. The U.S. Centers for Disease Control and Prevention (US CDC) and four of its implementing partners—International Medical Corps, the International Organization for Migration, RTI International, and the World Health Organization—worked in collaboration with the Government of Guinea to strengthen the country's surveillance capacity, in alignment with the Global Health Security Agenda and International Health Regulations 2005 objectives for surveillance and reporting. This paper describes the main surveillance activities supported by US CDC and its partners between 2015 and 2019 and provides information on the strategies used and the impact of activities. It also discusses lessons learned for building sustainable capacity and infrastructure for disease surveillance and reporting in similar resource-limited settings.

## Introduction

The 2014–2016 West Africa Ebola epidemic exposed major weaknesses in the global capacity to prevent, detect, and respond to infectious disease threats, and highlighted the need for reliable and sensitive disease surveillance systems. The Ebola outbreak started in December 2013 in Guinea's southeastern Guéckédou district but remained undetected until March 2014. By then, multiple chains of transmission had spread across the nearby porous borders to Sierra Leone and Liberia (1–3). The four-month delay between the index case and notification of the outbreak to national and international authorities, combined with lack of effective surveillance at the community level and points of entry, allowed for transboundary transmission and the escalation of the epidemic. A deficit of public health workers adequately trained in Integrated Disease Surveillance and Response (IDSR) (4), particularly at the peripheral level of the health system, and a largely paper-based system for collecting and aggregating disease data, contributed to underreporting of cases and problems with data completeness, accuracy, and reliability (5, 6).

To prevent future epidemics, the government of Guinea emphasized surveillance strengthening as a top priority in their National Health Development Plan, noting that at the time of the outbreak there was no functional system for epidemiological surveillance or alerts (7). Establishing core capacities for surveillance and reporting are critical action areas under the Global Health Security Agenda (GHSA), a multilateral and multi-sectoral initiative to accelerate progress toward implementation of the International Health Regulations 2005 (IHR) and enhance global and countries' capacities to prevent, detect, and respond to infectious disease threats, which Guinea joined in 2016 (8).

To achieve its strategic vision for enhancing surveillance of priority infectious diseases, the government of Guinea requested support from the U.S. Centers for Disease Control and Prevention (US CDC) and other partners (9, 10). In response, US CDC provided technical assistance directly and through a consortium of four implementing partners: RTI International (RTI), International Medical Corps (IMC), the International Office of Migration (IOM), and the World Health Organization (WHO). This article describes the primary surveillance activities of US CDC and its funded implementing partners and the lessons learned from the five-year engagement (2015–2019) with the government of Guinea to build sustainable systems and capacity for surveillance and reporting of priority infectious diseases.

## Methods

### Strategic planning and coordination

To ensure that surveillance activities aligned with the needs and priorities of the Guinean government, US CDC and its implementing partners worked directly with the MoH's National Health Security Agency (ANSS), the lead government agency in charge of epidemic preparedness and response in Guinea. The four overall objectives for surveillance strengthening, derived from the GHSA targets for surveillance and reporting of priority diseases and public health emergencies, were: (1) Improve sensitivity and specificity of the surveillance system; (2) Implement surveillance activities according to international norms; (3) Establish electronic reporting systems for surveillance and laboratory; and (4) Build capacity for management of surveillance data. To accomplish the surveillance strengthening objectives, the US CDC identified four primary activities (Table 1), strategically selected to align with the priorities outlined by the government of Guinea in its 2015 post-Ebola recovery plan (11) and to maximize impact by providing cross-cutting technical assistance and resources across objectives. For each strategic activity, the project partners collaborated with the ANSS and US CDC to develop actionable steps to advance and measure progress for accomplishing the surveillance strengthening objectives. Recognizing the fundamental importance of the workforce in the successful implementation of all these efforts, support for surveillance workforce development was implemented through integrating training and human resources capacity building throughout each of the strategic activities. Likewise, project partners ensured that information and communication technology infrastructure was established where needed, particularly with respect to strengthening community-based surveillance (CBS) and establishing electronic reporting.

**Table 1:** Strategic activities for surveillance system strengthening in guinea with implementing partners, 2015–2019.

	**Implementing partner**			
**Surveillance strategic activities**	**IMC**	**IOM**	**RTI**	**WHO**
Strengthening community-based surveillance	X	X	X	X
Reinforcing surveillance at points of entry		X		
Strengthening capacity for Integrated Disease Surveillance and Response (IDSR)			X	X
Establishing electronic reporting through DHIS 2			X	

*DHIS 2 indicates District Health Information Software 2; IMC, International Medical Corps; IOM, International Organization for Migration; RTI, RTI International; and WHO, World Health Organization*.

For each strategic activity, US CDC and its implementing partners consulted with the ANSS and relevant divisions of the MoH to determine high priority sub-activities and tasks. In the case of CBS, where multiple partners were involved in implementation, the MoH requested that each partner target a primary, discrete geographical area of intervention, to avoid duplication and ensure that technical assistance was well-distributed. Similarly, for the roll-out of electronic reporting, the MoH advised partners to pilot test the intervention at the district or regional level, with scale-up to the national level once shown to be effective and implementable (12). For each activity, an operational plan was developed to describe partner roles and responsibilities, goals and objectives, pre-implementation needs (e.g., pilot activities, pre-tests, etc.), implementation activities, indicators, monitoring and evaluation, resource needs and allocation, and timeline. A sustainability plan was incorporated into each operational plan in 2018. Each partner was responsible for developing the operational plan for its specific activities, in partnership with the MoH, ANSS, US CDC, and when appropriate, other contributing organizations. The plans were presented to the MoH and ANSS for review and final approval.

To further promote coordination and efficient use of resources, weekly meetings and stakeholder workshops were held for each of the strategic activities, to incorporate input and review objectives with other organizations engaged in the strategic activity, and refine the operational plans as needed. The partners also engaged in strategic planning activities with the government of Guinea to establish cohesive national guidelines and policies for achieving the objectives of GHSA and other relevant international initiatives, such as One Health. Indeed, the implementing partners benefited from the collaborative synergy of the One Health Platform, a multisectoral coordinating mechanism established by the Government of Guinea in April 2017 to foster collaboration and coordination among stakeholders from the human, animal, and environmental health sectors (3, 13). The emergence and reemergence of high-profile zoonotic diseases, notably Ebola in 2014–2016 and COVID-19 in 2020, speaks to the importance of a cross-disciplinary approach to address shared health threats at the human-animal-environment interface (14). Indeed, up to 75% of new or emerging infectious diseases globally are zoonotic in origin (15). Recognizing that early detection of zoonotic pathogens is imperative for preventing and controlling zoonotic disease threats, the GHSA directly references One Health in its goal to accelerate progress toward full compliance with the IHR 2005 and ensure that all countries in the world can detect, assess, report, and respond to emerging public health events of international concern, including outbreaks of zoonotic diseases (3) With this in mind, considerations for One Health were included in the strategic planning of each activity.

### Surveillance strategic activities

#### Strengthening community-based surveillance

**Figure 1:**
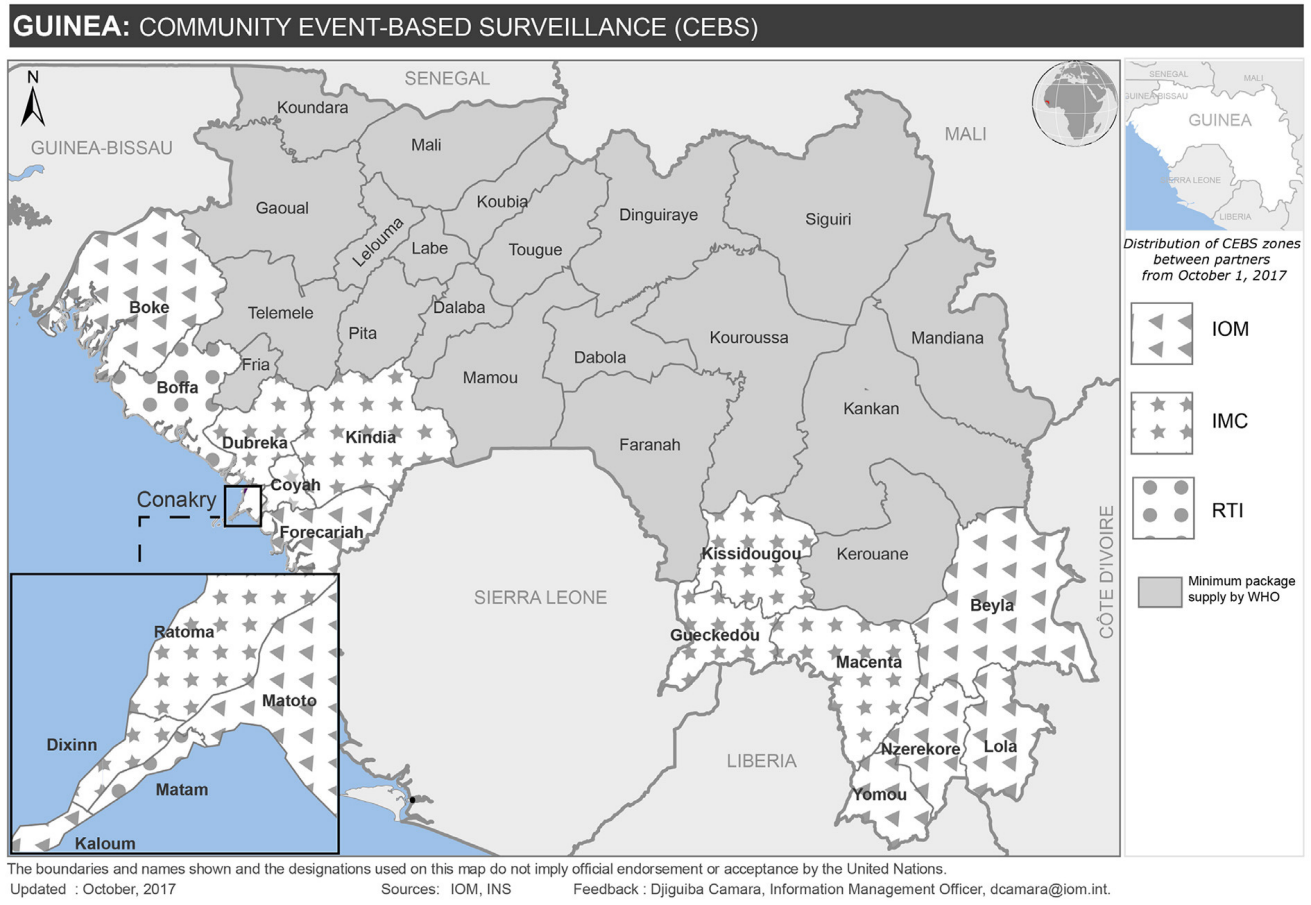
Distribution of US CDC partners implementing CBS in Guinea, 2015–2019.

In 2015, the MoH sponsored a national workshop to discuss priority activities to strengthen surveillance in the post-Ebola transition. The workshop revealed that community engagement was the greatest systematic weakness (16). As a result, the MoH launched a strategy for strengthening CBS of epidemic-prone diseases in priority prefectures that were considered at high risk of epidemics (10, 12). Building on lessons learned during the Ebola outbreak, during which the lack of community engagement resulted in delayed detection and under-reporting that thwarted early response efforts (2), the post-Ebola strategy focused on creating a strong network of trusted and well-trained community health workers to serve as the first line for disease detection and provide a critical link between the community and formal health services. Indeed, establishing a reliable, competent, and motivated public health workforce at the community-level as a critical component of the revised IDSR strategy (6). To achieve this goal, the MoH engaged a wide network of partners, including the US CDC implementing partners, to provide technical and financial support for a minimal package of activities and supportive materials. The partners positioned themselves in different geographical areas of intervention, based on where they had existing projects and local relationships, with RTI, IMC, and IOM providing coverage in 18 of Guinea's 38 health districts and WHO providing overall technical support (Figure 1)^1^. Examples of supportive activities included identification and training/re-training of community health workers; provision of equipment (such as phones and bicycles) to health centers and community health workers; support for communication costs; capacity building of community health workers in activities related to health promotion and behavior change; assistance with supervision of activities; and monitoring and evaluation.

Partners worked together to standardize implementation, particularly in areas where more than one US CDC implementing partner was present. For example, in Boffa district, where IOM and RTI were each responsible for four sub-districts, the partners worked together with the district health office to coordinate a common approach for the identification and training of community health workers, implementation, and supervision of activities. WHO facilitated a technical working group for the implementing partners to develop standard community case definitions and reporting tools for CBS activities across the country, and coordinated with the MoH to ensure that CBS activities were in alignment with its needs and priorities.

#### Reinforcing surveillance at points of entry

At the time that the first Ebola case emerged in Guinea in December 2013, cross-border surveillance systems were non-functional. During the Ebola response, surveillance at 34 land and maritime points of entry (PoE) were strengthened and mechanisms for cross-border surveillance were established. Recognizing that further investments would be needed to sustain these improvements and prevent future transmission at points of entry, the government of Guinea collaborated with IOM to build on the infrastructure developed during the Ebola response and create additional tools for continued cross-border surveillance. As a foundation, surveillance systems were established at points of entry to collect health and mobility data. To better understand vulnerabilities and risks at the borders and identify targeted strategies to reduce cross-border transmission, IOM implemented public health risk mapping activities. Using participatory mapping activities to identify areas at high-risk for transmission of epidemic-prone diseases, IOM facilitated group discussions with community stakeholders in prefectures sharing land or sea borders with neighboring countries to gather information about cross-border and in-country population flow and congregation spaces. This information was then mapped against epidemiological data to further assess public health vulnerabilities and risks at the border and along internal migration routes. IOM conducted these activities in collaboration with the MoH and the National Institute of Statistics to strengthen national capacity to conduct future population risk mapping for public health interventions. In addition, IOM organized cross-border meetings and built tools to exchange and share public health information between Guinea and its neighboring countries. Working with the IHR national focal point in the ANSS, IOM worked with US CDC, the African Field Epidemiology Network (AFENET) and RTI to enhance cross-border surveillance between neighboring districts in Guinea and Côte d'Ivoire, by facilitating cross-border coordination and data sharing between graduates of the Field Epidemiology Training Program in each country. The development and implementation of the Field Epidemiology Training Program in both countries was also supported by the US CDC, and thus demonstrates a strong example of collaboration across workforce development and surveillance technical assistance areas, both within Guinea and between neighboring countries. Finally, IOM also provided targeted training to health workers in border districts to improve detection and reporting of cross-border public health threats.

#### Strengthening capacity for IDSR

The IDSR framework, revised in 2010 to harmonize with the core capacity requirements under the IHR, increases the availability, quality, and usability of surveillance and laboratory data, helping health workers and decision-makers improve detection and response to public health threats. Guinea's national IDSR guidelines had not been reviewed since 2011 and did not contain locally relevant details regarding priority diseases and events, case identification, sample collection and testing, or notification procedures. In 2015, the MoH recognized IDSR strengthening as a priority activity for improving disease surveillance in the post-Ebola transition (11). To support this strategic objective, US CDC and the implementing partners engaged with the MoH and ANSS to build IDSR capacity at all levels (sub-district, district, regional, and national) of the health system. RTI and WHO led the IDSR strengthening activities, with technical input from the other implementing partners.

In February 2016, US CDC partnered with WHO to provide training on the IDSR technical guidelines. While reinforcing IDSR concepts, the training provided an opportunity to engage directly with the public health workforce responsible for IDSR implementation. Through observation and discussion with the trainees, several concerns about the 2011 version were identified: (1) the broad list of diseases did not reflect Guinea's priority diseases; (2) the case definitions were too complicated for health workers at all levels of the system; and (3) the procedures were too general, and did not reflect Guinea's health system structure, processes, or resources. As a result, the MoH engaged US CDC and its implementing partners to update the Guinea-specific IDSR technical guidelines, develop updated, standardized, and simplified case notification forms, and create job aids, such as standard operating procedures, to assist health workers at all levels of the system to appropriately implement IDSR activities.

Through engagement with US CDC, MoH, ANSS, US CDC's implementing partners and other surveillance stakeholders, WHO facilitated the evaluation of Guinea's 2011 IDSR technical guidelines and developed recommendations for a simplified guide focused on the priority diseases and events identified by the Guinean government. For each priority disease and event, the generic case definitions and IDSR procedures from the 2011 guidelines were adapted for the Guinean context. The revised guidelines were then reviewed and validated by all relevant stakeholders. This approach fostered country ownership and promoted a contextually appropriate IDSR system by actively engaging government leaders and critical stakeholders in the IDSR adaptation process (6). In August 2016, WHO helped produce and disseminate the simplified guidelines. To improve implementation of the revised IDSR guidelines, WHO also created a training curriculum for health workers. Where relevant, disease-specific technical support was also provided. For example, to support the addition of influenza to Guinea's IDSR technical guidelines, WHO assisted the MoH to develop and implement a plan for influenza sentinel surveillance. These activities aligned with a 2017 assessment of IDSR capacity among WHO Africa Member States, which compared data from 2014 and 2017 for two IDSR key performance indicators. The assessment showed increased IDSR capacity in Guinea, with on-going IDSR implementation covering ≥ 90% of subnational levels in 2017 compared to 50–89% coverage in 2014. There were also improvements in health workforce capacity, with ≥ 90% of health workers at all levels trained in the adapted IDSR guidelines in 2017 compared to ≥ 50% in 2014 (6). Although the direct impact of the IDSR strengthening activities cannot be measured, it is likely that they contributed to the enabling environment that made the improvements possible.

In 2017, in recognition that early detection of zoonotic pathogens is imperative for preventing and controlling zoonotic disease threats, RTI helped organize and facilitate a series of workshops between the MoH, the Ministries of Livestock and Environment, US CDC, the National Reference Laboratory, and other surveillance and laboratory stakeholders and partners to review and update the Guinea-specific IDSR priority diseases using a One Health approach (17). This multi-sectoral approach was intended to foster an enabling environment for collaboration and information sharing between the human, animal, and environmental health sectors, providing a foundation for future integration and interoperability of One Health data systems, as complementary reporting tools, structures, and systems for animal and environmental health became more advanced.

To further promote collaboration, RTI established a technical working group comprised of representatives from each ministry, US CDC, and the surveillance and laboratory partners, to review and revise the case notification forms for each of the defined priority diseases and events. To improve effective usage and uptake of the revised forms throughout the health system, the technical working group meetings, to which staff responsible for IDSR activities at the different levels of the health system were invited, were an opportunity for RTI to solicit feedback from health workers at the national, regional, and district levels, to ensure that the form was structured to follow the logical flow of data collection and availability of data at each point. Once feedback was collated, RTI integrated the comments into revised drafts of the forms, which were shared with MoH, ANSS, and sub-national stakeholders for final validation and approval.

In August 2018, US CDC and its implementing partners collaborated with the government of Guinea, including ANSS, MoH, and the Ministries of Agriculture and Environment, to launch nationwide training of trainers for the new case notification forms and the use of DHIS 2 (see following section) to manage, report, analyze, and present data. Nationwide scale-up of the new case notification forms and DHIS 2 began in June 2019, with complete coverage of Guinea's 38 health districts by May 2020. To reinforce the application of IDSR methodology among the public health workforce, RTI partnered with the Field Epidemiology Training Program to include of IDSR tools in their Frontline training curriculum and mentoring activities.

#### Establishing electronic reporting through DHIS 2

In 2015, as part of its long-term strategy for strengthening surveillance of epidemic-prone diseases, the government of Guinea identified the need for a comprehensive, national, electronic disease surveillance system and improved data management and analysis at all levels of the health system (9). This converged with the broader MoH initiative to strengthen the national health information system for monthly routine surveillance and reporting. To address this need, the US CDC implementing partners collaborated with the MoH and other surveillance stakeholders to assess the existing systems and information and communications technology (ICT) infrastructure, with the goal of identifying an appropriate platform and the ICT requirements for establishing a national electronic health information system. Critical ICT resources acquired during the Ebola response, such as laptop computers, desktop computers, and video projectors, were leveraged where available. When additional equipment was required, the implementing partners focused on purchasing high-quality, durable, context appropriate materials and engaged MoH staff throughout the process of installation. To facilitate local ownership and skill development, RTI trained MoH staff to serve as a resource for solar power and computer maintenance and support. Standard operating procedures and tools were developed to streamline the maintenance and repair process and introduce a mechanism for accountability and quality assurance.

The government of Guinea, through consultation with RTI and other implementing partners involved with health information systems strengthening, identified an open-source platform known as the District Health Information Software Version 2 (DHIS 2) to use for disease surveillance and reporting (18). DHIS 2 is a proven and robust health management information system platform, used in over 73 low-and-middle-income countries, with a user-friendly and highly-adaptable interface that can be easily tailored to enable integrated reporting and analysis of aggregate and case-based surveillance data across diseases. With a large international community of practice, including neighboring countries such as Sierra Leone and Liberia, DHIS 2 facilitates cross-border data-sharing and provides access to a wide network of users for technical support (12, 19). The Government of Guinea selected DHIS 2 as an integrated system for both routine monthly health reporting and epidemiological surveillance of epidemic prone diseases, thereby improving the sustainability of the overall system and eliminating the need to maintain parallel systems (12). The partners met regularly to formulate a strategy for DHIS 2 configuration and implementation. This included dividing responsibility for providing internet connectivity and follow up technical support, thereby alleviating the financial barriers associated with start-up.

Using the DHIS 2 platform as a foundation, RTI led the development and configuration of an epidemiologic surveillance module for weekly aggregated reporting and individual case notifications of the IDSR priority diseases and events, known as “SurvEpi”, distinct from the use of DHIS 2 for routine monthly health surveillance. RTI identified system requirements and configured the system through engagement with staff from the ANSS, the surveillance implementing partners (WHO, IMC, and IOM), US CDC, the national laboratories, and divisions/departments of the MoH with a role in disease surveillance and reporting^2^. In preparation for nationwide scale-up, the disease surveillance system was piloted in two regions (Boké and Labé) from June to October 2017. RTI collaborated with US CDC, the surveillance implementing partners, MoH, ANSS, and the Hemorrhagic Fever and Virology Laboratory (the national reference laboratory for most viral diseases) to evaluate the disease surveillance system pilot prior to nation-wide roll-out. In January 2018, SurvEpi was rolled out to all regions and districts in the country for aggregate weekly reporting. Following the development of revised IDSR case reporting forms, RTI worked closely with the MoH, WHO, IMC, Georgetown University (US CDC implementing partner for laboratory strengthening), and other surveillance and laboratory partners to configure the revised forms for individual case reporting in DHIS 2. During the IDSR training of trainers in August 2018, participants were also trained on the use of DHIS 2 for electronic individual case reporting.

To further promote the sustainability of DHIS 2 as the national platform for disease surveillance and advance surveillance workforce capabilities, RTI created partnerships with existing public health training programs. To reach frontline health workers, RTI developed a training module for inclusion in the Field Epidemiology Training Program curriculum. The module focused on the use of DHIS 2 for disease surveillance, reporting, and analysis. Using the success of university collaborations in other countries as a model (20, 21), RTI, the MoH and Guinea's national university, the Gamal Abdel Nasser University of Conakry, also initiated a partnership in July 2018 to further sustain the adoption of DHIS 2. This partnership aims to strengthen capacity within the university for DHIS 2 use and management by (1) engaging MoH staff to train students in the Masters of Public Health program on the use of DHIS 2 for management and analysis of disease surveillance data as part of the standard curriculum; and (2) engaging faculty and students from the computer sciences department in DHIS 2 configuration, maintenance and application development.

## Findings

### Strengthening community-based surveillance

The US CDC surveillance partners have helped strengthen early case detection by training more than 10,300 community health workers in 18 health districts to identify and report unusual health events and suspected cases of priority diseases from their community. The community health workers are supported by supervisors at 196 health facilities, who have received training and supportive equipment, such as telephones and motorcycles, to facilitate regular field visits and outbreak investigation. To enable rapid and consistent communication, the community health workers are equipped with mobile phones, SIM cards, monthly phone credits, solar chargers, and bicycles. Furthermore, the US CDC partners collaborated with the community health workers and health facility staff to improve awareness of epidemic-prone diseases, promote trust in health authorities, and encourage disease reporting through engagement activities such as health education chats (“causeries”) (*N* = 165, led by IOM), theater forums (*N* = 206, led by IOM), mass sensitization *via* radio and sketches (*N* = 2, led by RTI), community meetings (*N* = 93, led by RTI [*n* = 2] and IOM [*n* = 91]; IOM's community meetings focused on cross-border issues), and monthly meetings involving local leaders and health agents (*N* =18, led by IMC).

The standardized procedures and tools developed collaboratively by the CDC partners and the MoH have been systematically deployed throughout the country to facilitate timely reporting to the closest health facility, then to district health offices for further investigation. The community-level data have been integrated into weekly surveillance and individual case reporting of priority diseases that is reported to the national level. In 2019, 126 alerts were made by community health workers that were verified and thus resulted in a case-notification form entered into DHIS 2: 86 for measles/rubella; 17 for meningitis; 12 for rabies; four for acute flaccid paralysis; three for icteric syndrome; two for maternal and neo-natal tetanus; and one each for influenza-like illness and viral hemorrhagic fever syndrome.

### Reinforcing surveillance at points of entry

**Figure 2:**
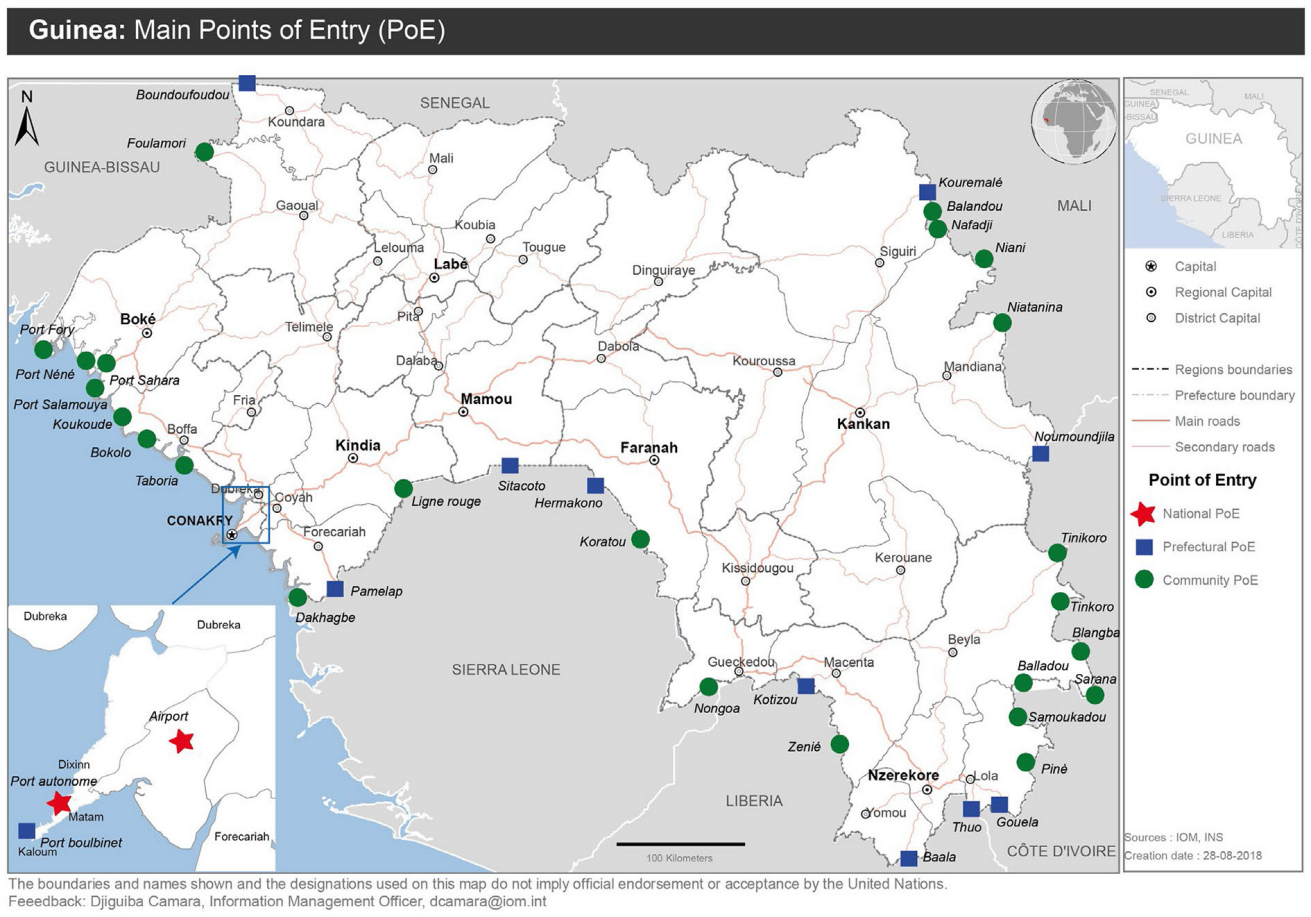
Points of entry targeted for surveillance strengthening, 2015–2019.

IOM reinforced capacity at 41 points of entry by developing and validating a national protocol for cross-border surveillance (Figure 2). Training was provided to 644 surveillance agents in border areas on best practices for detecting and reporting disease threats, and protection kits were distributed, consisting of items such as identification jackets, boots, hand sanitizers, examination gloves, face masks, hygiene kits, and other related items. The protocol was tested through a series of simulations in the format of tabletop exercises and drills. IOM helped develop and test a response plan for health emergencies at Guinea's Conakry International Airport. Through participatory mapping of public health risks in 115 of 341 sub-districts within 23 districts with border areas, IOM helped identify 8,080 sites vulnerable to infectious disease threats and mapped 527 health structures near the border and 634 formal and informal entry points for target surveillance strengthening activities. During the participatory mapping activities, IOM established mechanisms for sustainability by training 160 local surveillance agents in participatory mapping techniques and 3,520 community leaders to identify vulnerable sites. To build community awareness of cross-border transmission and protective actions, IOM leveraged its network of 4,126 community health workers to sensitize 54,261 community members in 12 health districts along the borders of Guinea-Bissau, Senegal, Sierra Leone, Liberia, and the Ivory Coast. Additionally, 242 volunteers were trained in health control measures at 15 maritime border points of entry (Conakry, Dubréka, Forécariah, Boffa and Boké harbors and landing stages).

### Strengthening capacities for IDSR

Through consensus discussions with stakeholders, 13 priority diseases and events were identified for weekly epidemiological surveillance and reporting (Table 2). Malaria is also reported weekly in the aggregate report but not individually *via* case notification forms.

**Table 2:** Guinea-specific IDSR priority diseases and events with differential diagnosis (2017).

**Type of disease/event**	**Name of disease/event**	**Differential diagnosis**
**Epidemic Prone Diseases**	Viral hemorrhagic fever syndrome	Ebola; yellow fever; Marburg; Lassa fever; Rift Valley fever; dengue fever
	Icteric fever syndrome (yellow fever)	Yellow fever; hepatitis; leptospirosis; Congo Crimean fever; Rift Valley fever;
		dengue fever
	Influenza-like illnesses	Seasonal flu; avian flu; swine flu; influenza new sub-type
	Acute diarrheal syndrome (cholera)	Cholera; shigelloses; rotavirus; collective food poisoning
	Measles	Rubella
	Meningococcal meningitis	Non-meningococcal meningitis
**Diseases targeted for eradication**	Acute flaccid paralysis (polio)	Wild poliovirus; Vaccine-derived poliovirus
	Neonatal/Maternal tetanus	N/A
**Zoonotic diseases**	Anthrax	N/A
	Rabies	N/A
	Brucellosis	N/A
**Other events of public health importance**	Maternal deaths	N/A
	Adverse events following vaccination	N/A

Case reporting forms for these 13 priority diseases and events were streamlined and standardized to promote accurate application of standard case definitions, adherence to IDSR technical guidelines, and integration of data from clinical and laboratory sources. Each form was revised to include an agreed-upon data collection structure that is consistent with IDSR guidelines. Standardized sections were applied across forms for case identification, notification, hospitalization, actions taken, and feedback tracking. The revised forms include disease-specific information on risk factors, signs and symptoms, and laboratory analysis and results. The forms use a logical data collection flow that tracks the patient's information from the facility at which they first present to higher levels of care (as required), up to the national level. Laboratory test results for the patient are also integrated into the same case report form, to further facilitate data integration and completeness. In addition, supportive job aids for laboratory and surveillance staff at every level of the health pyramid were developed to improve application of IDSR guidelines and promote proper use of the revised case reporting forms. The revised forms have been incorporated into the national DHIS 2 electronic surveillance system, which allows data entry at the district, regional, national, and laboratory level and supports rapid and complete reporting.

The IDSR and DHIS 2 training of trainers produced a cadre of 55 trainers, representing the participating ministries, national reference laboratories, and critical surveillance partners. From August 2018 to May 2019, these trainers provided comprehensive IDSR (combined with DHIS 2, as described in the following section) training to health workers nationwide, using a cascade approach in which each level of the pyramidal structure of the MoH trains the next level. The training also included instruction on safe and secure sample collection, handling and referral, provided *via* IMC and Georgetown University, in line with the concurrent development and implementation of Guinea's national specimen referral policy (22). Overall, more than 1,230 health surveillance workers at the national, regional, district, and sub-district level across all of Guinea's 38 health districts were trained on the use of the new IDSR case notification forms to support improved management, reporting, analysis, and decision-making. National scale-up of DHIS 2 for individual case reporting and management of IDSR priority diseases began in June 2019 and had reached complete coverage of Guinea's 38 health districts by March 2020. The results of the national scale-up are reported by Reynolds et al. (12).

The integration of IDSR and DHIS 2 into Field Epidemiology Training Program curriculum and mentoring activities helped to reinforce implementation of the tools. A 2018 evaluation of Guinea's Field Epidemiology Training Program reported that graduates of the training program for frontline workers were able to identify the necessary procedures and tools to conduct disease surveillance tasks at their level, including data collection, reporting, analysis and evidence-based decision-making (23). Although this evaluation was conducted before the completion of IDSR and DHIS 2 scale-up, it suggests that the synergy between these three activities can be leveraged to increase uptake and reinforce effective use of IDSR tools, including DHIS 2. It will be important to continue periodic evaluation to assess whether knowledge and proper application of IDSR tools is sustained.

### Establishing electronic reporting through DHIS 2

US CDC and its implementing partners helped develop a realistic and sustainable information technology strategy for electronic disease surveillance and reporting at the district and regional levels, focused on provision of essential, context-appropriate software, equipment and mechanisms for building local capacity to maintain the ICT infrastructure. RTI provided basic computer skills training to staff operating in project-supported health districts. RTI supported ICT infrastructure development in all districts in the Boké and Labé Regions and three health districts in Conakry (Matam, Dixinn, and Ratoma), plus two national-level reference laboratories and the ANSS. To ensure that computers provided to health facilities and offices remained functional and protected against viruses, the local IT maintenance service providers and RTI staff mentored local focal points who could assist in managing maintenance requests and troubleshooting minor issues. The plan developed by RTI in collaboration with the MOH focused on quarterly preventative computer maintenance. RTI conducted routine supervision visits to provide on-site support and to confirm with other beneficiaries at the health facility that their computers were functioning as expected. This process allowed rapid identification and response to issues by providing more intensive support, supplemental training or assistance with troubleshooting and resolving equipment issues.

Through the SurvEpi module in DHIS 2, health workers at the district, regional, and national level can collect, manage, analyze, report and make decisions based on case data for Guinea's 13 priority diseases, conditions, and events identified for immediate and weekly reporting by the Ministries of Health, Livestock, and Environment (12). The six-month pilot testing phase of DHIS 2 for disease surveillance was evaluated positively by the MoH (data not shown) (12). In March 2018, RTI completed nationwide scale-up of DHIS 2 for weekly aggregated case reporting (i.e., “case counts”), laying the groundwork for individual case reporting *via* DHIS 2. By the end of May 2019, 388 health workers nationwide at the national, regional, and district level were trained on the use of DHIS 2 for electronic case reporting. Through the SurvEpi module, the capacity has been established for real-time surveillance and reporting at health offices and hospitals in Guinea's 38 health districts, as well as all 40 public health diagnostic laboratories at the district, regional and national levels. The nationwide system makes it possible for health workers at all levels of the system to access and analyze weekly disease surveillance data, disaggregated to the facility level, as soon as it is entered into the system. In addition, authorized health workers can access and analyze individual case notification data through the system. To enable more efficient reporting and resolution of issues related to DHIS 2, RTI set up an online helpdesk system using MantisBT (version 2.4.0) open source software: https://support.sante.gov.gn/login_page.php.

The system has also proven to be quickly adaptable to emerging disease threats. In 2020, less than a month after the first case of COVID-19 was confirmed in Guinea, the ANSS swiftly launched a COVID-19 module for case-level surveillance (12, 19). In addition to individual COVID-19 case-reporting, stakeholders at all levels, including laboratories, were able to view case characteristics, produce summary statistics, and access a real-time dashboard with data visualization features for evidence-based decision making (12). When a new Ebola outbreak was reported in February 2021, DHIS 2 was again adapted and deployed within 3 days of the first case report, allowing stakeholders at the national and district level to capture and manage information on cases, contacts, alerts, laboratory results, and vaccinations (19).

As a result of the partnership agreement with MoH, the Gamal Abdel Nasser University of Conakry, and RTI, RTI renovated and equipped computer laboratories at the university to enable students and faculty to work on DHIS 2 in and outside of classes. In addition, RTI helped establish a steering committee for MoH and university representatives to manage and oversee partnership activities. The first cohort of public health students received training in August 2018. RTI collaborated with the MoH to conduct a DHIS 2 workshop for 30 students who wanted to learn how to use the software for public health data management and analysis. The workshop provided an overview of the health information system, and what data are collected and why. Specific training sessions focused on entering aggregate and individual case data and analyzing data using DHIS 2 built-in analysis tools including pivot tables, data visualizer and mapping applications. Furthermore, RTI sponsored a faculty member from the university to attend a one-week workshop on DHIS 2 server management offered by the West Africa Health Organization (WAHO) through the West Africa Health Informatics Team (WAHIT) initiative, with technical assistance from the University of Oslo. During the workshop, staff from MoH, ANSS, and the Gamal Abdel Nasser University of Conakry were trained on DHIS 2 configuration and management.

## Discussion

Since 2015, CDC and its implementing partners have worked closely with the government of Guinea, and specifically the MoH, to improve surveillance capabilities, per Guinea's commitments under the IHR and in alignment with the GHSA. The progress and outcomes of these efforts have resulted in a number of lessons learned and recommendations, which may be helpful to inform future surveillance strengthening efforts in Guinea, or indeed similar programs in other resource-limited settings (Table 3).

**Table 3:** Lessons learned and recommendations from implementation of surveillance capacity strengthening activities, 2015–2019.

**Strategic activity**	**Lesson(s) learned**	**Recommendation(s)**
**Cross-cutting**	Highly collaborative work processes, involving multiple partners and stakeholders, can be time-consuming but result in a highly quality, more locally-adapted, and widely accepted product. Planning for the transition of activities is crucial; unexpected changes in partner funding can jeopardize the sustainability of a program if long-term planning has not taken place in a timely way. Frequent communication and effective coordination between implementing partners can help realize opportunities for leveraging investments, as well as overcome identified barriers.	➢ Err on the side of inclusiveness when conducting stakeholder engagement. ➢ Implementation timelines should account for the time needed for stakeholder engagement, consensus-building, and iterative review of key documents/plans. ➢ Transition planning, including explicit consideration of sustainability, should take place as early as possible in the project planning cycle, and not left until the final year of a project. ➢ Funders, and national government stakeholders, should explicitly encourage implementing partners to establish formal coordination mechanisms to align efforts, such as joint implementation plans, regular meetings, etc.
**Community-based surveillance**	The Ebola outbreak response in Guinea relied heavily on community volunteers, who could then be trained/re-trained to support other surveillance functions. Community health workers that supported other health activities before the Ebola outbreak provided a synergistic pool of personnel to support surveillance. Empowering community health workers may be able to amplify other efforts to promote community awareness of disease or social mobilization. Community engagement is critical for building trust and awareness within the community and facilitates willingness to report potential cases of disease or unusual events.	➢ Where possible, implementation approaches that leverage past activities are more likely to be accepted by the community, attract suitable candidates for training, and be effective. ➢ Efforts should be made to integrate CBS into on-going health activities, so as not to create parallel health structures at community level, nor overburden community health workers. ➢ Future CBS activities should seek to include indicators on community knowledge, attitudes, and practices related to health seeking behavior and reticence, to better measure this potential secondary impact of CBS. ➢ To maximize community engagement, strategies should leverage buy-in from a wide network of trusted community members and develop a diversity of formal and informal communication mechanisms and data sources for raising awareness and expanding the availability of information to detect unusual events of potential public health risk that have not been picked up through routine indicator-based surveillance
**Strengthening surveillance at points of entry**	The lack of legal documents and national policy orientations for cross-border collaboration is a hindrance to the implementation of public health activities involving neighboring countries. The identification and selection of surveillance officers from border communities is a factor of success and sustainability of surveillance at points of entries.	➢ Countries contemplating points of entry surveillance should envision a national framework of strategies to guide cross-border activities. ➢ The One Health concept should be applied for successful points of entry surveillance under the leadership of the Ministry of Health, without undermining the involvement of other border management stakeholders including the Ministry of Territorial Administration. ➢ Identification of objective selection criteria of surveillance officers and the involvement of national partners in health are success factors in the implementation of points of entry surveillance. ➢ Ensuring that communities provide inputs in health security activity at all stages and discussing sustainability early in the process will foster ownership and sustainability in a context where communities receive a great amount of development and health funding from multiple sources.
**IDSR strengthening**	The One Health concept provided a mechanism for engaging the animal and environmental health sectors in disease surveillance and response activities, resulted in a more comprehensive list of priority diseases and accompanying guidelines, and established a foundation for on-going multisectoral collaboration and information-sharing. IDSR is an ideal framework for bringing together the laboratory and surveillance sectors and coordinating training efforts.	➢ Where possible, multisectoral approaches to strengthening disease surveillance and response can result in leveraging a wider pool of expertise (and funding sources), promote earlier recognition of emerging public health threats, and lead to more effective disease control.
**Establishing electronic disease reporting**	Linking training on DHIS 2 as the platform for electronic disease surveillance with IDSR highlighted the complementarity between these efforts and prevented duplication of effort and “workshop fatigue” among participants When newly established capacities rely on technology and infrastructure, ensuring local experts are trained to provide troubleshooting and maintenance will enhance operability and sustainability.	➢ As part of the coordination process, implementing partners should regularly review upcoming training and other activities, to identify where harmonization can occur. ➢ National universities and existing training programs are a valuable source of local talent and should be engaged to develop local experts.

US CDC implementing partners leveraged the community engagement activities initiated during the Ebola response in 2014 into an effective approach for CBS. Through their extensive network of community health workers, the US CDC partners have helped create a rapid response mechanism to provide much-needed support to the MoH during outbreaks. For example, community health workers have since been involved during outbreaks of yellow fever, polio, measles and meningitis to assist with active surveillance and vaccination campaigns. In select sites, they have been trained to support sentinel surveillance of cholera.

Importantly, the impacts of implementing CBS are not restricted to epidemic-prone diseases. Community health workers in Guinea have previously been engaged in other health activities such as malaria prevention, vaccination campaigns, and family planning (24–26). The additional resources provided by partners supporting community health workers for epidemic-prone disease surveillance may thus help, indirectly or directly, to sustain these other health activities, although efforts must be taken to ensure coordination, and that community health workers do not become overburdened. It is also worth noting that the intensive community engagement conducted as part of the CBS efforts may have served to amplify other on-going efforts to promote community awareness and engage community members through social mobilization, and helped to overcome community reticence and hostility, although future work is needed to better characterize potential linkages between CBS activities and community trust.

In turn, while initially set up as an emergency effort to support the Ebola response, CBS activities have now been integrated into Guinea's long-term surveillance strengthening strategy. By March 2019, the US CDC surveillance partners had ended their activities and transferred financial and operational ownership to the MoH, and thus played an integral role in developing the national strategy for transitioning CBS activities to the MoH (16). The plan aimed to integrate CBS more fully into prefectural annual operational plans and budgets, including income generating activities for sustaining community health workers, CBS activities, and necessary material and equipment. The Transition Plan was approved and validated in November 2018, by the MoH's National Division of Community Health, the ANSS, US CDC and all local partners involved in CBS activities. The partners have worked together to harmonize the CBS tools, materials, and approach outlined in the transition strategy, using lessons learned from each partner to maximize the effectiveness and potential sustainability. This includes consideration of supportive equipment and materials–essentially a “CBS toolkit”–to foster timely and effective detection and reporting of suspected cases or unusual events, such as bicycles, solar panels, mobile phones, chargers, and SIM cards. When planning CBS activities, future implementers should ensure that supportive materials and equipment are appropriate to the local context. Further, the value of seemingly small investments should not be overlooked. For example, in Guinea, many communities lack paved roads and rainy season lasts 4–6 months, community health workers were equipped with bicycles and raincoats. This low-technology solution was cost-effective, sustainable, and added significant value by making it easier and faster for community health workers to move within and between communities to detect potential disease threats and then report them to near-by health centers.

Community engagement was also seen to have impacts beyond CBS. Engagement of communities in border regions can also positively impact efforts to strengthen surveillance and other health security capacities at points of entry. Indeed, selection of border health agents from those communities can facilitate communication, coordination, and understanding, enhancing the likelihood of timely detection of possible health security threats at border crossings. Despite the existence of regional frameworks guiding overall surveillance and response efforts, such as IDSR, the lack of policies or other guidelines specifically for cross-border activities proved to be a challenge with respect to improving surveillance at points of entry. National frameworks for cross-border collaboration, that are harmonized regionally for alignment and translatability, would help to overcome these barriers, and could have impact beyond the health sector, particularly if developed with a One Health approach in mind.

One Health was an important element integrated into the development of revised case notification forms, which demonstrated an effective, collaborative, multisectoral approach to IDSR. Bringing together the Ministries of Health, Livestock, and Environment led to the inclusion of three high-priority zoonotic diseases (anthrax, rabies, and brucellosis) that were previously not under surveillance, as well as recommendations for enhancing surveillance for the existing priority zoonotic diseases, such as influenza and Ebola. This process has thus provided the government of Guinea with a framework for strengthening surveillance of its five zoonotic diseases of greatest public health concern, which enables measurement of progress toward achieving the objectives of the Zoonotic Disease technical area under the IHR's Joint External Evaluation, part of its Monitoring and Evaluation Framework (27). It has also helped establish a foundation for on-going and future collaboration across ministries using a One Health approach to strengthen Guinea's national health surveillance system. While IDSR activities have previously focused heavily on building capacity for human disease surveillance, it is critical that similar attention is given to animal health. Although direct activities to strengthen animal surveillance fell outside of this project's scope, the focus on One Health helped establish an enabling environment for improved data-sharing between the human and animal sectors, including multisectoral consensus on focal priority zoonotic diseases, identification of preliminary set of common data elements for human and animal health, and the integration of focal points from the each of the relevant ministries into weekly surveillance meetings at the national and district level. As electronic surveillance systems for animal and environmental health are further developed, these enabling factors can be leveraged for future integration and interoperability of human, animal, and environmental surveillance data. The MoH and surveillance partners should continue to work with the Ministries of Livestock and Environment to build surveillance capacity for detecting and controlling zoonotic threats while they are still in animal populations and to develop compatible human and animal surveillance data fields within DHIS 2 for more efficient, integrated data systems, accompanied by necessary ICT training and support for livestock and environment officers at the district, regional, and national levels.

Further, the collaboration among implementing partners and across sectors contributed to a more efficient process and strengthened the development and implementation of the IDSR tools and their integration into the national DHIS 2 platform. The implementing partners brought complementary technical skills, increased available resources, and expanded the reach of the activities to each partner's specific network of collaborators, which helped facilitate buy-in from different parts of the MoH, as well as other ministries and local stakeholders. For example, the trainings on the revised case notification forms and DHIS 2 implementation leveraged RTI's technical expertise in epidemiology, data management, and analysis and IMC's technical expertise in specimen management and laboratory diagnosis. The computer maintenance skills, solar panels, and other ICT infrastructure and support provided by RTI, WHO and other partners promoted greater longevity of the computers used for IDSR data collection, and also critical to implementation of DHIS 2.

The combination of IDSR and DHIS 2 training was efficient as both are key elements of the surveillance system and target the same end users. Similarly, the involvement of diverse partners in the pilot evaluation provided a chance to hear directly from DHIS 2 users and stakeholders at the district, regional, and national level, which facilitated targeted improvements to the system and procedures for electronic case management and reporting. For example, feedback from the users led to inclusion of data fields to enable laboratories to enter their data in the aggregate weekly reports. However, successful implementation of both IDSR and DHIS 2, as well as surveillance efforts more broadly, will rely on effectively training health workers throughout the system and providing on-going supportive supervision (6). In order to sustain the investments in surveillance system strengthening, local partners need to invest in building in-country capacity to maintain and support the system long-term without dependence on international technical assistance. The mechanisms identified by the implementing partners to link activities to the curricula of existing training programs are an example of this kind of continuity. For example, engaging the Field Epidemiology Training Program reinforce IDSR methodology and to use and promote DHIS 2 as the national tool for disease surveillance data reporting and analysis increases uptake and effective use of the system, while providing training and support for management and analysis of surveillance data. The partnership with the Gamal Abdel Nasser University of Conakry also promotes long-term sustainability by providing continuous training to rotating cohorts of public health students and future professionals, as well as provide a way for the university to expand professional opportunities to faculty and students as part of the DHIS 2 international community. Since the launch of the DHIS 2 partnership with the university, the MoH, RTI, and additional partners have expressed interest in providing financial and technical support to sustain this initiative. For example, the MoH has received additional financial support to hire a consultant to develop a plan to design and implement a formal curriculum on DHIS 2 into the computer sciences department at the university. This type of institutional capacity-building has the potential for far-reaching and long-lasting impact. As public health professionals and health workers serve as a critical frontline safeguard against emerging disease threats, the impact can be further amplified by expanding the curriculum to include explicit training in IDSR methodology and tools (6, 28).

The emergence of COVID-19 in 2020 and a reemergence of Ebola in 2021 has provided a real-world test of Guinea's IDSR and DHIS 2 capacity building investments. Less than a month after the first COVID-19 case was confirmed in Guinea, DHIS2 was adapted and deployed at the national and district level to manage the country's response to the pandemic (12, 19). The equally rapid response to a new Ebola outbreak in February 2021, in the midst of the on-going COVID pandemic, suggests that DHIS 2 is a flexible and resilient system. It will be important to monitor the continued performance of DHIS 2 (e.g., data completeness and timeliness) for on-going IDSR, as well as COVID-19 and Ebola-specific surveillance and response to determine whether these systems truly resilient in the face of new and emerging disease threats. Global analytical frameworks such as the GHS Index, an assessment and benchmarking of health security and related capabilities across the 195 countries, provide a valuable tool for understanding the existing capacity for the prevention and mitigation of emerging public health threats and measuring performance over time (29). The 2021 GHS Index shows that Guinea is ranked 156/195, with an overall score of 26.8, indicating that significant and sustained investments are needed to address gaps and strengthen epidemic and pandemic preparedness. Among the indicators relevant to the surveillance strengthening activities described in this paper (zoonotic disease, real-time surveillance and reporting, surveillance data accessibility and transparency, case-based investigation, and epidemiology workforce), no progress has been made from 2019 to 2021 (29). This may be explained by the additional and unexpected pressure on the Guinean health system and public health workforce caused by the COVID-19 pandemic and the likely diversion of financial and human resources to higher-priority areas such as clinical care. Effective and sustained health security capacity depends on political will and government readiness and flexibility to direct available resources to evidence-based public health recommendations for disease containment and mitigation (29). The government of Guinea should continue to use the GHS Index to monitor its performance overtime to identify risk factors and capacity gaps that can help inform technical strategies and financial commitments to establish sustained health security capacity.

## Conclusion

Since 2015, US CDC and its implementing partners have helped the Government of Guinea shape and implement a cohesive strategy for establishing a sustained and timely system for epidemic-prone disease surveillance and reporting. Through the strong relationships forged with the MoH and engagement at all levels of surveillance system, US CDC and its partners have provided technical support for development of complementary tools and procedures for the foundational surveillance activities, including CBS, enhanced surveillance at points of entry, IDSR strengthening, and real-time surveillance and reporting using DHIS 2. This harmonized and collaborative approach promotes complete, accurate, and timely data from the community to the national level and has also helped to establish important linkages across sectors, through engagement with key laboratory stakeholders and *via* One Health. The surveillance partners' technical expertise and close collaboration with the MoH and ANSS played a vital role in moving Guinea closer to achieving the IHR and GHSA targets for surveillance and reporting; in April 2017, Guinea underwent the Joint External Evaluation process, and the mission report cited the use of DHIS 2 as a strength of the surveillance system (30). By employing a proactive approach to transition planning, including concrete and explicit consideration of sustainability throughout the project lifecycle, the surveillance partners fostered an enabling environment for sustained surveillance capacity improvements and helped prepare the government of Guinea to take financial, operational, and technical ownership at the project's end. The model of partnership, and corresponding impact of the projects described here to epidemic-prone disease detection, reporting, and response in Guinea, can provide useful lessons learned for building surveillance capacity and infrastructure in similar settings.

## Data availability statement

The original contributions presented in the study are included in the article/supplementary material, further inquiries can be directed to the corresponding author.

## Author contributions

JH-F, LM, CS, and PM conceptualized the manuscript. JH-F, BD, SB, SK, LM, ST, AW, MW, and PM oversaw management of the described projects. JH-F, BD, ID, MBB, MB, AK, LK, MK, ER, OS, KS, and AW contributed to implementation of project activities and collection of relevant data. JH-F and CS led development of the first draft. SC and SB drafted their organizations' contributions to the manuscript. DC provided all mapping support and figure development. LM coordinated the clearance process. All authors reviewed drafts, provided edits, and approved the final version.

## Funding

This publication was supported by Cooperative Agreements U19GH001591 (RTI), 1U2GGH001761 (IOM), and 1U19GH001587-01 (IMC), funded by the U.S. Centers for Disease Control and Prevention.

## Conflict of interest

The authors declare that the research was conducted in the absence of any commercial or financial relationships that could be construed as a potential conflict of interest.

## Publisher's note

All claims expressed in this article are solely those of the authors and do not necessarily represent those of their affiliated organizations, or those of the publisher, the editors and the reviewers. Any product that may be evaluated in this article, or claim that may be made by its manufacturer, is not guaranteed or endorsed by the publisher.

## Author disclaimer

Its contents are solely the responsibility of the authors and do not necessarily represent the official views of the Centers for Disease Control and Prevention or the Department of Health and Human Services.

## Footnotes

^1^Note that “Community Event-Based Surveillance (CEBS) was the terminology used when the project was initially being implemented.

^2^These included: *Institute National de Santé Publique* [National Public Health Institute]; *Bureau de Stratégie et Développement* [Office of Strategy and Development); *Bureau de Système National d'Information Sanitaire* [National Health Information System Office]; *Département de Prévention et Lutte Contre la Maladie* [Department of Disease Prevention and Treatment]; *Programme Élargi de Vaccination* [Expanded Vaccination Program]; *Programme National de Lutte Contre le Paludisme* [National Malaria Program]; *Département National de Sante Familiale* [National Department on Family Health]; Select surveillance staff from the Regional and District Health Departments, hospitals, and health centers.

## References

[1] Bell BP Damon IK Jernigan DB Kenyon TA Nichol ST O'Connor JP. Overview, control strategies, and lessons learned in the CDC response to the 2014–2016 ebola epidemic. MMWR Suppl. (2016) 65:4–11. 10.15585/mmwr.su6503a2 27389903

[2] Dahl BA Kinzer MH Raghunathan PL Christie A De Cock KM Mahoney F. CDC's Response to the 2014–2016 ebola epidemic — guinea, liberia, and sierra LEONE. MMWR Suppl. (2016) 65:12–20. 10.15585/mmwr.su6503a3 27388930

[3] Standley CJ Carlin EP Sorrell EM Barry AM Bile E Diakite AS. Assessing health systems in Guinea for prevention and control of priority zoonotic diseases: a One Health approach. One Heal. (2019) 7:100093. 10.1016/J.ONEHLT.2019.100093 31049389 PMC6479159

[4] WHO CDC. Technical Guidelines for Integrated Disease Surveillance and Response. (2010). Available online at: http://www.afro.who.int/sites/default/files/2017-06/IDSR-Technical-Guidelines_Final_2010_0.pdf (accessed May 27, 2020).

[5] Hemingway-Foday J Souare O Reynolds E Dialio B Bah M Karamokoba Kaba A. Improving Integrated Disease Surveillance and Response Capacity in Guinea, 2015-2018. Online J Public Health Inform. (2019) 11. 10.5210/ojphi.v11i1.9837

[6] Fall IS Rajatonirina S Yahaya AA Zabulon Y Nsubuga P Nanyunja M. Integrated Disease Surveillance and Response (IDSR) strategy: Current status, challenges and perspectives for the future in Africa. BMJ Glob Heal. (2019) 4:e001427. 10.1136/bmjgh-2019-001427 31354972 PMC6615866

[7] Ministère de la Santé. Plan National de Développement Sanitaire (PNDS) 2015-2024. Conakry (2015). Available onlin at: http://extwprlegs1.fao.org/docs/pdf/gui158099.pdf

[8] Global Health Security Agenda. A Partnership Against Global Health Threats. Available online at: https://ghsagenda.org/ [Accessed January 27, 2020]

[9] Ministère de la Santé. Plan de Renforcement de la Surveillance des Maladies à Potentiel Epidémique en Guinée (2015-2017). Conakry: Ministère de la Santé (2015).

[10] Marston BJ Dokubo EK van Steelandt A Martel L Williams D Hersey S. Ebola Response Impact on Public Health Programs, West Africa, 2014–2017. Emerg Infect Dis. (2017) 23: 10.3201/eid2313.170727 PMC5711323 29155674

[11] Ministère de la Santé. Plan de relance du système de santé (2015-2017). Conakry: Ministère de la Santé (2015).

[12] Reynolds E Martel LD Bah MO Bah M Bah MB Boubacar B. Implementation of DHIS2 for disease surveillance in Guinea: 2015-2020. Front Public Health. (2021) 9:761196. 10.3389/fpubh.2021.761196 35127614 PMC8811041

[13] Agbo S Gbaguidi L Biliyar C Sylla S Fahnbulleh M Dogba J. Establishing National Multisectoral Coordination and collaboration mechanisms to prevent, detect, and respond to public health threats in Guinea, Liberia, and Sierra Leone 2016-2018. One Health Outlook. (2019) 1:4. 10.1186/s42522-019-0004-z 33829125 PMC7990095

[14] Technical guidelines for integrated disease surveillance response in the African region: Third edition. WHO | Regional Office for Africa. Available online at: https://www.afro.who.int/publications/technical-guidelines-integrated-disease-surveillance-and-response-african-region-third (accessed June 21, 2022).

[15] Salyer SJ Silver R Simone K Barton Behravesh C. Prioritizing zoonoses for global health capacity building—themes from one health zoonotic disease workshops in 7 countries, 2014–2016. Emerg Infect Dis. (2017) 23. 10.3201/eid2313.170418 PMC5711306 29155664

[16] Ministère de la Santé. Plan de Transition de la Surveillance Base Communautaire (Draft). Conakry: Ministère de la Santé (2018). p. 13.

[17] Standley CJ MacDonald PDM Attal-Juncqua A Barry AM Bile EC Collins DL. Leveraging partnerships to maximize global health security improvements in Guinea, 2015-2019. Heal Secur. (2020) 18:S34–S42. 10.1089/hs.2019.0089 32004131 PMC11323542

[18] Health Information Systems Program. District Health Information Software Version 2 (DHIS 2). (2018) Available online at: https://www.dhis2.org/ (accessed June 26, 2019).

[19] Correction: Implementing a DHIS2 Ebola virus disease module during the 2021 Guinea Ebola outbreak. BMJ Glob Health. (2022) 7:e009240corr1. 10.1136/bmjgh-2022-009240corr1 35654449 PMC9163536

[20] Karuri J Waiganjo P Orwa D Manya A. DHIS2: the tool to improve health data demand and use in Kenya. J Health Inform Dev Ctries. (2014) 8:38–60.

[21] Manoj S Wijekoon A Dharmawardhana M Wijesuriya D Rodrigo S Hewapathirana R. Implementation of district health information software 2 (DHIS2) in Sri Lanka. Sri Lanka J Bio-Medical Informatics. (2012) 3:109–14. 10.4038/sljbmi.v3i4.5431

[22] Standley CJ Muhayangabo R Bah MS Barry AM Bile E Fischer JE. Creating a National specimen referral system in guinea: lessons from initial development and implementation. Front Public Heal. (2019) 7:83. 10.3389/fpubh.2019.00083 31111025 PMC6499205

[23] Collins D Diallo BI Bah MB Bah M Standley CJ Corvil S. Evaluation of the first two Frontline cohorts of the field epidemiology training program in Guinea, West Africa. Hum Resour Health [Internet]. (2022) 20:40. 35549712 10.1186/s12960-022-00729-w PMC9097411

[24] Plucinski MM Guilavogui T Sidikiba S Diakité N Diakité S Dioubaté M. Effect of the Ebola-virus-disease epidemic on malaria case management in Guinea, 2014: a cross-sectional survey of health facilities. Lancet Infect Dis. (2015) 15:1017–23. 10.1016/S1473-3099(15)00061-4 26116183 PMC4669675

[25] Delamou A Koivogui A Dubourg D Delvaux T. Family planning in Guinea: a need for better public commitment. Trop Med Int Heal. (2014) 19:65–73. 10.1111/tmi.12219 24175994

[26] Ciglenecki I Sakoba K Luquero FJ Heile M Itama C Mengel M. Feasibility of mass vaccination campaign with oral cholera vaccines in response to an outbreak in Guinea. PLoS Med. (2013) 10:e1001512. 10.1371/journal.pmed.1001512 24058301 PMC3769208

[27] WHO. Joint external evaluation tool: International Health Regulations (2005) second edition. Geneva. (2018). Available online at: http://www.who.int/ihr/publications/WHO_HSE_GCR_2018_2/en/ (accessed May 27, 2020).

[28] Ravi SJ Meyer D Cameron E Nalabandian M Pervaiz B Nuzzo JB. Establishing a theoretical foundation for measuring global health security: a scoping review. BMC Public Health. (2019) 19:954. 10.1186/s12889-019-7216-0 31315597 PMC6637489

[29] Ghsindex.org. Available online at: https://www.ghsindex.org/wp-content/uploads/2021/12/2021_GHSindexFullReport_Final.pdf (accessed June 19, 2022).

[30] World Health Organization. Evaluation Externe Conjointe des Principales Capacités RSI de la République de Guinée. Rapport de mission: 23-28 avril, 2017. (2017). Available online at: https://apps.who.int/iris/bitstream/handle/10665/258726/WHO-WHE-CPI-REP-2017.40-fre.pdf?sequence=1&isAllowed=y (accessed October 17, 2019).

